# Body representation in dreams of congenital and early-life amputees

**DOI:** 10.1038/s41598-024-83000-7

**Published:** 2025-03-22

**Authors:** Martin Diers, Michael Schredl, Herta Flor, Robin Bekrater-Bodmann

**Affiliations:** 1 Institute of Cognitive and Clinical Neuroscience, Central Institute of Mental Health, Medical Faculty Mannheim, Heidelberg University, Mannheim, Germany; 2 Clinical and Experimental Behavioral Medicine, Department of Psychosomatic Medicine and Psychotherapy, LWL University Hospital, Ruhr University Bochum, Alexandrinenstrasse 1-3, 44791, Bochum, Germany; 3 Sleep Laboratory, Central Institute of Mental Health, Medical Faculty Mannheim, Heidelberg University, Mannheim, Germany; 4 Department of Health Science and Technology, Center for Neuroplasticity and Pain (CNAP), SMI ®, Aalborg University, Aalborg, Denmark; 5 Department of Psychosomatic Medicine and Psychotherapy, Central Institute of Mental Health, Medical Faculty Mannheim, Heidelberg University, Mannheim, Germany; 6 Department of Psychiatry, Psychotherapy and Psychosomatics, Uniklinik RWTH Aachen, Aachen, Germany; 7 Scientific Center for Neuropathic Pain Aachen SCN AACHEN, Uniklinik RWTH Aachen, Aachen, Germany

**Keywords:** Amputation, Congenital limb deficiency, Amelia, Dreams, Phantom limb pain, Medical research, Neurology

## Abstract

Phantom limb pain (PLP) is a common consequence of the amputation of a limb. Individuals with congenital limb absence (here: congenital amputees), however, seem to rarely experience PLP. Previous results suggest that the experience of PLP in the waking state affects the recalled body appearance in dreams of individuals with acquired limb amputation, with PLP being associated with the recall of an impaired rather than an intact body. However, it remains unclear how congenital amputees – who never experienced an intact body and rarely PLP – recall their body appearance in dreams. In the present cross-sectional study, we assessed body-related dream content in a sample of adult congenital amputees and compared their reports with those from adult persons with an acquired limb amputation early in life. We found that congenital amputees reported the least frequent dreams with an intact body, and after birth, the age at amputation positively predicted the recall of an intact body in dreams. The effects were not explained by time since amputation and the presence or absence of PLP. This suggests that life experiences of an intact body find expression in self-related dream content.

## Introduction

After the amputation of a limb, most amputees report the presence of a phantom limb, that is, the perception of the missing body part. The phantom limb can be accompanied by non-painful or painful experiences, the latter which referred to as phantom limb pain (PLP). While 48.5% of amputees who lost their limb after the age of 6 report the presence of PLP^1^, the prevalence in person who were amputated before the age of 6 is with 15 to 20.5% significantly lower^1,2^.

People who are born with a amelia (i.e., congenital limb absence, here referred to as congenital amputation) have the lowest prevalence of phantom experiences including PLP^2^. Current etiological models of PLP emphasize the influence of peripheral, spinal, and brain changes after limb amputation which, however, does not or at least to lesser degree occur in early-life or congenital amputees^3–7^.

Based on the continuity hypothesis of dreaming – stating that experiences in the waking state are reflected in dream content^8^ – one would expect that an amputated body is accompanied by dreams characterized by a higher frequency of the own body being recalled as amputated rather than intact. To date, only few studies have investigated the representation of the dreamed body in amputees (for a review see^9^). In a large sample of 2,156 amputees, Bekrater-Bodmann et al.^10^ assessed the percentage of remembered dreams that featured an intact or impaired body representation of the dreamer. About 48% of the dreams featured an intact body (i.e., as prior to the amputation), whereas in about 14% of the dreams the body was impaired (i.e., as after the amputation), seemingly contradicting the continuity hypothesis of dreaming. However, there was a significant relationship between habitual PLP levels and the recall of an impaired body in dreams, suggesting that negative sensations related to the amputation experienced in the waking state (here: PLP) find their way into dreams, supporting the continuity hypothesis^8^. In a small diary study with *N* = 20 persons who recently lost a limb due to cancer, about 50% reported dreams with an intact body within three weeks to six months after surgery^11^. This longitudinal study emphasizes that dreams not only incorporate the current waking-life experiences but also rely on memory of the times before the amputation.

We were interested in assessing the body representation in dreams of congenital amputees. Such findings might help to disentangle whether only body experiences in waking life and/or (potentially) innate central body models^12^ affect dream content. In addition, including PLP into this analysis might help to differentiate between effects of the extent of previous experiences of having or not having an intact body and negative consequences of the amputation (here: PLP). Previous studies in blind individuals have indicated that those who lose their sight after the age of 7 years retain visual imagery in their dreams whereas those who are congenitally blind do not report visual features in their dreams^13,14^. For congenital amputees and those with an acquired amputation later in life, a similar finding regarding the body representation in dreams can be expected.

The present study assessed dream reports in congenital and early age amputees and assessed their body representation in dreams using a previously validated methodology^10^. Based on the evidence reviewed above, we hypothesized that the body representation in dreams (at least partly) depends on the lived experience with an intact body. We therefore assumed that congenital amputees are dreaming themselves very rarely or even not at all with an intact body (due to having no such waking-life experiences), and the frequency of an intact body representation in dreams will increase as a function of age at limb amputation. Since PLP has been shown to predict the body representation in the amputees’ dreams^10^, and since age at amputation predicts PLP^2^, we further included PLP in a regression analytical approach in order to control for potentially confounding effects.

## Results

### Sample description

From the total sample of *n* = 182 participants, *n* = 50 were in the con-uncorr group (congenital amputees without a limb correction later in life), *n* = 35 were in the 0-2y group (amputation between birth and 2 years of age), *n* = 34 were in the 3-4y group (amputation between 3 and 4 years of age), *n* = 38 were in the 5-6y group (amputation between 5 and 6 years of age), and *n* = 25 were in the con-corr group (congenital amputees with a limb correction later in life). Of this sample, *n* = 99 were male and *n* = 83 were female. The mean age at the time of assessment was 52.4 ± 15.5 years. The mean age at amputation was 1.4 ± 0.8 years in the 0-2y group, 3.5 ± 0.5 years in the 3-4y group, and 5.6 ± 0.5 years in the 5-6y group. An accident was the most frequently reported reason for acquired limb amputation. One hundred eleven participants reported upper limb amputation and 70 had a lower limb amputation (one missing value), with 89 being right-sided and 93 being left-sided amputees. On average, the participants lost about half of the respective limb (residual limb length of 53.8 ± 23.0%). One hundred eight participants used a prosthesis. Descriptive data on dream frequency and body representation in dreams can be found in Table 1.

**Table 1:** Descriptive data of congenital amputees without limb correction later in life (con-uncorr), subjects with an amputation at an age between birth and 2 years (0-2y), between 3 and 4 years (3-4y), and between 5 and 6 years (5-6y), as well as normative data of a sample with an amputation after the age of 18 years (18y+) and congenital amputees with a limb correction later in life (con-corr).

	Main groups				Sub- and normative groups	
	Congenital (con-uncorr)	0–2 years (0-2y)	3–4 years (3-4y)	5–6 years (5-6y)	Congenital with limb correction (con-corr)	Normative data 18y+
n	50	35	34	38	25	1533
Age (m ± std)	48.86 ± 15.2	47.9 ± 14.4	57.7 ± 14.8	54.1 ± 17.3	55.8 ± 13.0	63.8 ± 15.6
Age at amputation (m ± std)	---	1.4 ± 0.8	3.5 ± 0.5	5.6 ± 0.5	15.2 ± 10.7	36.1 ± 18.0
Sex (male/female)	24/26	21/14	23/11	20/18	11/14	1237/296
Deam frequency (n (%))						
Almost every morning	4 (8.0)	1 (2.9)	3 (8.8)	4 (10.5)	2 (8.0)	193 (12.6)
Several times a week	14 (28.0)	9 (25.7)	5 (14.7)	5 (13.2)	5 (20.0)	397 (25.9)
Once a week	12 (24.0)	7 (20.0)	3 (8.8)	7 (18.4)	6 (24.0)	288 (18.8)
2–3 times a month	9 (18.0)	10 (28.6)	3 (8.8)	8 (21.1)	3 (12.0)	250 (16.3)
Once a month	5 (10.0)	4 (11.4)	6 (17.6)	5 (13.2)	3 (12.0)	184 (12.0)
Less than once a month	6 (12.0)	4 (11.4)	14 (41.2)	9 (23.7)	6 (24.0)	221 (14.4)
Number of dreams in % in which a certain body representation occurred (m ± std)						
Impaired	10.20 ± 27.22	11.23 ± 29.53	15.62 ± 26.69	3.95 ± 8.07	8.92 ± 20.62	13.77 ± 23.62
Intact	19.90 ± 34.88	19.06 ± 34.91	35.03 ± 39.847	34.34 ± 41.67	26.08 ± 39.21	50.66 ± 40.11
Not remembered	69.90 ± 40.36	69.71 ± 42.72	49.29 ± 44.67	61.71 ± 42.38	65.00 ± 41.83	35.54 ± 39.70
Number of individuals who report an intact body representation in dreams (n (%))	15 (30.0)	11 (31.4)	19 (55.9)	20 (52.6)	12 (48.0)	1151 (75.1)
PLP prevalence (n (%) yes)	0 (0)	2 (5.7)	3 (8.8)	11 (28.9)	2 (8.0)	1023 (66.7)

m ± std = mean ± standard deviation.

### Factors associated with the body representation in dreams

**Fig. 1:**
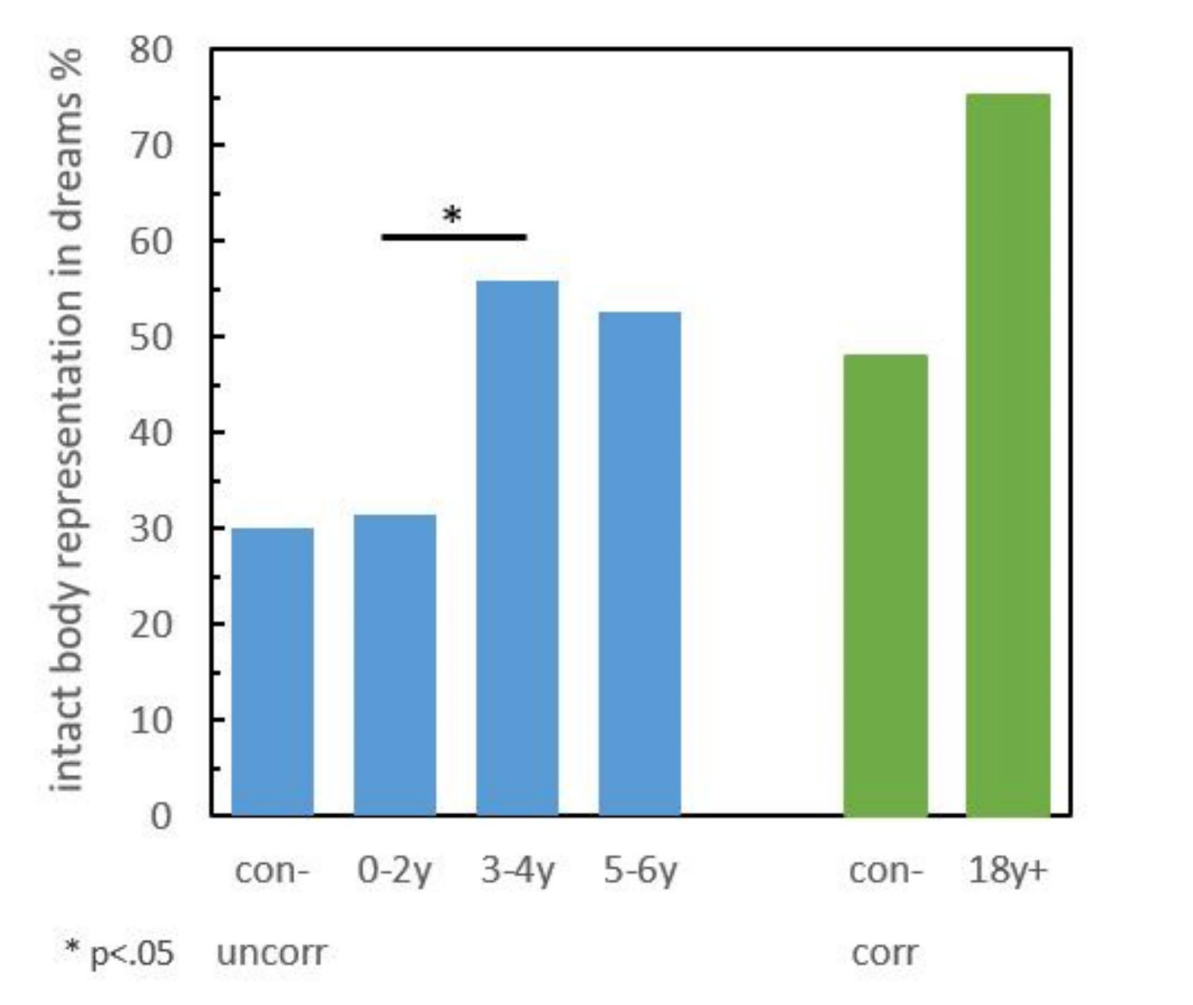
Percentage of persons with intact body representation in dreams (depicted in blue) for the groups congenital amputees without limb correction later in life (con-uncorr), subjects with an amputation at an age between birth and 2 years (0-2y), between 3 and 4 years (3-4y), and between 5 and 6 years (5-6y), as well as normative data with an amputation after the age of 18 years (18y+) and congenital amputees (depicted in green) with a limb correction later in life (con-corr); these group values are given only for descriptive purposes.

To determine the effect of age at amputation (factor group [con-uncorr, 0-2y, 3-4y, 5-6y]) on the intact body representation in dreams, we calculated a binomial logistic regression (model 1). The binomial logistic regression model including group was statistically significant, (χ^2^(1) = 7.166, *p* = .007; explained variance of Nagelkerke’s *R*^2^ = 0.06). Overall percentage of accuracy in classification was 59.9%, with a sensitivity of 30.8% and a specificity of 80.4%. The variable group contributed significantly to the recall of an intact body representation in dreams (*p* = .009). A higher age at amputation increased the likelihood of an intact body representation in dreams, OR = 1.460 (95%-CI [1.101, 1.934]). All model coefficients and odds ratios are provided in Table 2. The percentage of persons with intact body representation in dreams is presented in Fig. 1.

The additional analyses including PLP prevalence (model 2) and time since amputation (model 3) indicated no influence of the respective variables. In more detail, the binomial logistic regression model including group and PLP prevalence was statistically significant, (χ^2^(2) = 9.489, *p* = .009; explained variance of Nagelkerke’s *R*^2^ = 0.079). Overall percentage of accuracy in classification was 59.2%, with a sensitivity of 33.8% and a specificity of 77.2%. Only group (*p* = .046) significantly predicted intact body representation in dreams, while PLP prevalence (*p* = .138) showed no significant effect. A higher age at amputation increased the likelihood of an intact body representation in dreams, OR = 1.355 (95%-CI [1.006, 1.825]). Also, the model including group and time since amputation was statistically significant, (χ^2^(2) = 7.258, *p* = .027; explained variance of Nagelkerke’s *R*^2^ = 0.061). Overall percentage of accuracy in classification was 59.9%, with a sensitivity of 30.8% and a specificity of 80.4%. Again, only group (*p* = .008) significantly predicted intact body representation in dreams, while time since amputation (*p* = .761) showed no significant effect, and again, a higher age at amputation was associated with a higher frequency of an intact body representation in dreams, OR = 1.463 (95%-CI [1.103, 1.940]).

Post hoc tests revealed that the recall of an intact body representation in dreams was significantly different between the groups 0-2y and 3-4y (χ^2^(4) = 4.197, *p* = .041, Cramér’s V = 0.247), with the 3-4y group reporting higher recall of an intact body in dreams, but not significantly different between the groups con-uncorr and 0-2y (χ^2^(4) = 0.020, *p* = .888, Cramér’s V = 0.015) or the groups 3-4y and 5-6y (χ^2^(1) = 0.076, *p* = .782, Cramér’s V = 0.033).

**Table 2:** Model coefficients and odds for the three binomial logistic regressions with intact body representation in dreams being the criterion.

	B	SE	Wald	*p*	Odds ratio	95%-CI for odds ratio	
						Lower bound	Upper bound
Model 1							
Group	0.378	0.144	6.923	0.009	1.460	1.101	1.934
Constant	− 0.886	0.268	10.943	0.001	0.412		
Model 2							
Group	0.304	0.152	3.990	0.046	1.355	1.006	1.825
PLP	0.887	0.598	2.204	0.138	2.428	0.753	7.835
Constant	− 0.869	0.268	10.521	0.001	0.419		
Model 3							
Group	0.380	0.144	6.973	0.008	1.463	1.103	1.940
Time since amputation	− 0.003	0.011	0.092	0.761	0.997	0.976	1.018
Constant	− 0.729	0.581	1.573	0.210	0.483		

Degrees of freedom were 1 for all Wald statistics.

## Discussion

The results of the present study reveal a relationship between the age of amputation early in life and the recalled body representation in dreams. Specifically, age of amputation predicted the recall of an intact body, suggesting that life experiences of an intact body find expression in self-related dream content. Moreover, there seems to be a phase in infantile development (around the age of 2–3 years) where changes in the physical integrity might have long-lasting formative influences on an individual’s cognition. The effects cannot be attributed to the time since amputation and the presence of PLP, emphasizing the importance of neurotypical developmental processes for the establishment of a person’s internal body model.

Mulder et al.^12^ suggested the existence of a basic neural representation of the body that is, at least partly, genetically determined and might be relatively insensitive to changes in the body periphery as those caused by an amputation. Our finding that only 30% of the congenital amputees report dreams with an intact body representation seem to support this hypothesis. However, the results of our study indicate that experiencing limb loss in the waking state might play a superior role in determining body-related dream content compared to the hypothetical role of an innate body model, as the congenital amputees without limb correction reported a lower percentage of an intact body representation in dreams than congenitally amputated persons who underwent limb correction later in life.

The observed relationship between age at amputation and body-related dream content was independent of PLP. This is important, since a younger age at amputation has been associated with less PLP^2^, and less PLP has been associated with more dreams with intact body representation^10^. That this relationship does not hold for congenital and early-life amputees emphasizes the relevance of experiences an individual has made with their own body. Our data suggest that a relevant developmental process occurs between 2 and 3 years of age, since a significant increase in the recall of an intact body occurred (about 30% vs. about 50%). This effect might be related to the maturation of memory systems in the brain. Infantile or childhood amnesia refers to the adult’s relative paucity of episodic memories from early life phases^15,16^. It is assumed that adults fail to recall events that occurred prior to an average age of 3 years (estimated range 2–4 years)^16^ which fits nicely to our data. In accordance with the continuity hypothesis of dreaming^17^ and the idea that dreams are a reflection of our waking experiences, it is plausible that individuals who lost a limb prior to the age of 3 cannot explicitly remember themselves as intact. However, since we found low but not absent recalls of an intact body representation also in persons who lost their limb before the age of 3, our data suggest that implicit memory or other cognitive processes affect dream content. Interestingly, congenital amputees with a limb correction later in life showed a dream pattern similar to that of persons who lost their limb at the age of 3 or later, suggesting that the surgery might have activated certain memory processes that would not occur without limb correction. However, since we did not assess the nature of the congenital anomaly, it could also be that those who underwent limb correction had congenital malformations that are different from amelia.

The assumption that the lived sensorimotor experience predicts dream content is supported by results from congenitally blind persons, where similar observations were made. Thus, it has been shown that congenitally blind persons do not have any visual perceptions in their dreams^18^, while they report more frequently auditory, tactile, gustatory, and olfactory dream components compared to sighted controls^13^. In those who became blind later in life, duration of blindness was negatively correlated with duration, clarity, and color content of visual dream impressions^13^. In the body perception domain as assessed in the present study, however, we could show that the dream content is particularly driven by the age of amputation and not by the duration of living with an amputation, suggesting a manifestation of body representation early in life. Two studies in congenital paraplegics, however, found that these patients mainly recalled dreams in which their body was not disabled^19,20^. An interpretation could be that dream content draws information from innate body representations, a concept which was introduced by Melzack et al.^21^. Alternatively, there might be differential effects of distinct body representation (e.g., purely sensory vs. motor ones). Another interpretation is that sensing other persons with intact bodies in daily life and during media consumption might influence the dreams of amputated individuals. There is empirical evidence that media content affects dream content^22^. However, there might be multiple mechanisms that explain how dream content can include sensorimotor content that has never been experienced^20^. Another study showed that recently paralyzed participants reported more kinesthetic dream content than long-term paralyzed participants^23^, which is similar to the earlier reported inverse relationship between time since amputation and intact body recall in adult amputees^10^ applying a similar methodology as in the present study. Together, previous and our results emphasize that dreaming is not solely influenced by the recent past, but reverts to experiences made in the entire life^24^.

Our study has several weaknesses. First, we did not characterize the type of congenital malformations which might have provided additional valuable information. However, the central message of our paper is not influenced by this missing information. Second, we assessed the body representation in dreams only retrospectively which has a higher risk of bias for non-experience-based reports. A strength of our study is that we assessed dream recall frequency which strongly affects the results of dream reports^25^. This is an advantage compared to the study by Mulder et al.^12^ who did not consider dream recall frequency in their analysis, which could be problematic, as those with a low dream recall frequency may have difficulty in accurately reporting their dream content^25^. Furthermore, most studies mentioning the amputee’s body representation in dreams used rather coarse measures for recall assessment, probably biasing the results^9^. Future studies could validate the present retrospective results by waking up participants in the REM phase of sleeping in a laboratory setting. Further, prospective investigations could also better characterize the congenital malformations that were assessed solely by self-report in the present study.

## Materials and methods

### Study design and sample recruitment

This study included data from individuals with congenital or acquired limb loss who participated in a cross-sectional Germany-wide survey conducted as part of the European Research Council Advanced Grant PHANTOMMIND (“Phantom phenomena: A window to the mind and the brain”). The sample of the current study was also part of an earlier report describing the prevalence and characteristics of painful and non-painful phantom phenomena in limb amputees^26^, and a study specifically focusing at post-amputation pain in congenital and early-life amputees^2^; dream content in these groups, however, was not analyzed. All included participants gave written informed consent to take part in the study, and the Ethics Committee of the Medical Faculty Mannheim of Heidelberg University approved the protocol, which adhered to the Declaration of Helsinki. From our data base of 3,374 unilateral limb amputees^26^, 252 participants met the inclusion criteria of an congenital or early-life amputation.

### Assessment

The PHANTOMMIND survey included adapted core items of the Phantom Pain and Limb Phenomena Interview^27^, an interview on prosthesis use^28^, the West Haven-Yale Multidimensional Pain Inventory^29^ modified to separately assess PLP and residual limb pain. The survey consisted of 53 items in total, including drawings, divided into five parts: (A) demographic information, characteristics of the amputation, prosthesis and medication use, general physical and mental health and well-being; (B) measures of PLP; (C) measures of phantom limb sensations including referred sensations and telescoping; (D) measures of residual limb pain; and (E) measures of empathy, attitude towards transplantations, sleep, dreams (for details see below), and ethnicity^26^. In the present study, we focused on the pain and dream content data. There were analogous versions for arm and leg amputees. Current PLP intensity was assessed with a numerical rating scale from 0 = no perception to 10 = very strong perception.

### Dream questions

Dream recall frequency was recorded on a seven-point scale (coded as 0 = never, 1 = less than once a month, 2 = about once a month, 3 = two or three times a month, 4 = about once a week, 5 = several times a week, and 6 = almost every morning). Participants who reported a dream recall frequency of never (*n* = 53) or had missing data (*n* = 3) were excluded. Participants who responded affirmatively in the dream recall frequency scale (response > 0) were asked to indicate the distribution of recalled body representation in dreams on a percentage basis: “How are your dreams distributed among the following categories? Your indications should add up to 100%”. The three response categories were (a) impairment due to amputation is present in the dream, (b) the body in the dream is intact and no impairment occurred, and (c) perception of the own body in dreams could not be remembered. These data were only entered into statistical analyses when the added-up percentages had a deviation of a maximum of 1% from 100% due to minor rounding errors. Otherwise, the indications were implausible and suspected to be invalid. Data of *n* = 4 amputees did not meet this criterion and *n* = 10 had missing data in these items. Since we were specifically interested in the dreams with an intact body, only this data was considered in the statistical analyses.

### Data analysis

Statistical analyses were conducted using *IBM SPSS v*27 *(IBM*,* Armonk*,* NY*, USA). For this study, we focused on data of congenital amputees and subjects who underwent amputation up to the age of 6. Based on Melzack et al. et al.^21^ and Diers et al.^2^, we created four groups: congenital amputees (con), and persons with an amputation between birth and 2 years (0-2y), 3–4 years (3-4y), and 5–6 years (5-6y). Further, we split the congenital amputee group with respect to performed (con-corr) / not performed (con-uncorr) limb correction later in life, which might have an impact on the investigated relationships. A limb correction later in life refers to any surgically correction of the limb. As the age at which the limb correction was performed was highly heterogeneous, these data will only be reported descriptively.

For the statistical analysis, we focused on the recall of an intact body representation in dreams, which was transformed from percentage into binary values, with 0 for 0% and 1 for > 0%. We calculated a binomial logistic regression (model 1) to determine the effect of age of amputation (factor group [con, 0-2y, 3-4y, 5-6y]) on the intact body representation in dreams. To strengthen the main analysis, we have conducted two additional analyses to investigate the influence of potentially important variables. First, to disentangle the related effects of age at amputation, PLP, and dream content^2,10^, we performed a binomial logistic regression (model 2) where we additionally included PLP prevalence (0 for PLP absence and 1 for PLP intensities > 0). Second, to disentangle the related effects of age of amputation, time since amputation, and dream content, we performed another binomial logistic regression (model 3) where we additionally included time since amputation (in years). Post-hoc chi-square tests and Cramér’s V effect sizes were computed to compare the intact body representation in dreams between con and 0-2y groups, 0-2y and 3-4y groups, as well as 3-4y and 5-6y groups. For comparison purposes, we further present normative data of amputees with an amputation after the age of 18 years (18y+; data adapted from^26^).

## Data Availability

The data supporting the findings of this study is available from the corresponding author, Martin Diers, upon request.
